# ﻿*Monomoriumdine* sp. nov. (Hymenoptera, Formicidae): a new inquiline social parasite ant species from North America

**DOI:** 10.3897/zookeys.1243.145744

**Published:** 2025-06-25

**Authors:** Stefan P. Cover, Christian Rabeling

**Affiliations:** 1 Museum of Comparative Zoology, Harvard University, 26 Oxford Street, Cambridge, MA 02138, USA Harvard University Cambridge United States of America; 2 Department of Integrative Taxonomy of Insects, Institute of Biology, University of Hohenheim, Garbenstraße 30, 70599 Stuttgart, Germany University of Hohenheim Stuttgart Germany; 3 KomBioTa – Center for Biodiversity and Integrative Taxonomy Research, University of Hohenheim & State Museum of Natural History, Stuttgart, Germany University of Hohenheim & State Museum of Natural History Stuttgart Germany

**Keywords:** Brood parasitism, inquilinism, integrative taxonomy, Myrmicinae, Nearctic, social parasitism

## Abstract

Among the very rarest of Nearctic ants are three species of inquiline social parasites belonging to the genus *Monomorium*, namely *Monomoriuminquilinum* DuBois, *Monomoriumpergandei* (Emery), and *Monomoriumtalbotae* DuBois. All three species are known only from the type collections. Here, we describe *Monomoriumdine* Cover & Rabeling, **sp. nov.**, from the Navajo Nation in New Mexico, USA, a new species closely similar to the three known social parasites. Like them, *M.dine* appears to be a workerless inquiline that exploits a free-living *Monomorium* host. We also provide keys to the queens of the Nearctic *Monomorium* inquilines, provide the first images of these species, report new collections for *Monomoriumtalbotae* DuBois, discuss host-parasite associations, and summarize what is presently known about these mysterious social parasites.

## ﻿Introduction

The ant genus *Monomorium* (Hymenoptera, Formicidae, Myrmicinae) is globally distributed, comprising almost 300 described, extant species (Bolton 2024). It is especially abundant and speciose in the Old World Tropics, with centers of diversity in the Afrotropical region and in Australia (Ettershank 1966; Heterick 2001, 2006). By comparison, the New World *Monomorium* fauna is miniscule. There are 11 described free-living and social-parasitic species native to North America, Mexico, and the Caribbean region (DuBois 1986; Fisher and Cover 2007; Seifert 2025). Until recently, all North American species belonged to the *Monomoriumminimum* group (DuBois 1986; Bolton 1987; Fisher and Cover 2007), but a fresh taxonomic revision of this group recognizes *Monomoriumminimum* (Buckley) as a junior synonym of *Monomoriumcarbonarium* (Smith) (Seifert 2025). According to this new revision, the *M.carbonarium* group now consists of 10 free-living species. Eight are native to North America. One exotic, *M.carbonarium*, has almost certainly been introduced to Europe from eastern North America, and two species are native to Europe (Seifert 2025). The social-parasitic species were not reviewed. In the New World, seven additional species have been recorded from South America, which are only distantly related to the North American species (Fernández 2007; Ward et al. 2015; D. Gotzek unpublished data).

A molecular phylogenetic study of *Monomorium* and relatives in the subfamily Myrmicinae (Ward et al. 2015) resolved some problems on the generic level but, except for the global pest *Monomoriumpharaonis* Linnaeus, we know comparatively little concerning the taxonomy, ecology, and life-history traits within the genus as a whole. While the vast majority of *Monomorium* species are free-living, six apparent social parasites have been described (DuBois 1986; Bolton 1987). *Monomoriuminquilinum* DuBois, *Monomoriumpergandei* (Emery), and *Monomoriumtalbotae* DuBois occur in the Nearctic (Emery 1893; DuBois 1981, 1986), whereas the other three parasite species occur in the Old World (Bolton 1987). The North American social parasites are astonishingly rare. Presently, all three species are known from the type collections only (DuBois 1986). All appear to be inquiline social parasites that live within the nests of their hosts throughout their entire life cycles, except for a brief dispersal period. In general, inquiline ant females seek adoption in host colonies where they usurp the brood care behavior of the host and substitute the production of the host sexual offspring with their own (reviewed by Wilson 1971; Buschinger 2009; Rabeling 2021). Most inquilines have lost the worker caste entirely and produce sexual offspring only. Despite their highly specialized life histories, inquiline social parasites have evolved multiple times convergently across the ant tree of life (Gray and Rabeling 2023).

Here, we describe a new, fourth *Monomorium* inquiline species from North America. This new species was collected by Gary Alpert as part of an ant biodiversity inventory of the Navajo Nation (https://www.antwiki.org/wiki/Ants_of_the_Navajo_Reservation). In addition, we provide a key to the New World *Monomorium* social parasites, report some new information about host associations and biogeography of previously described *Monomorium* social parasites, and discuss our findings in the context of *Monomorium* social parasite biology.

## ﻿Materials and methods

### ﻿Specimens examined

In addition to new collections, specimens from the insect collections listed below were examined for this study:

**CRC** C. Rabeling Collection, University of Hohenheim, Stuttgart, Germany

**LACM** Los Angeles County Museum of Natural History, Los Angeles, CA, USA

**MCZC** Museum of Comparative Zoology, Harvard University, Cambridge, MA, USA

### ﻿Morphometric measurements

Specimens were examined and measured using a Leica MS5 stereomicroscope fitted with a stage micrometer. Measurements were taken at 100× magnification. Morphometric conventions and indices follow Bolton (1987) and modifications described by Cover and Rabeling (2024). Morphometric measurements and indices are defined as follows:

**HL** Head length. Length of the head in full face view, excluding mandibles, measured in a straight line from the midpoint of the anterior clypeal margin to the midpoint of the posterior margin of the head. In species where the posterior margin or the clypeal margin (or both) is concave, the measurement is taken from the midpoint of a transverse line spanning the anteriormost or posteriormost projecting points respectively.

**HW** Head width. Maximum width of head, not including the eye.

**CI** Cephalic index. HW × 100/HL

**SL** Scape length. Maximum straight-line length of the antennal scape excluding the basal constriction or neck close to the condylar bulb.

**SI** Scape index. SL × 100/HW

**ML** Mesosoma length. Diagonal length of the mesosoma in profile from the point at which the pronotum meets the cervical shield to the posterior base of the metapleuron.

## ﻿Results

### ﻿Key to the queens of *Monomorium* social parasite species occurring in the Nearctic region including Mexico

**Table:** 

1	Individuals large, relatively robust, gaster larger than mesosoma (ML 0.88–1.90); anterior border of clypeus with distinct central emargination flanked by two prominent teeth, mandibles well developed, broadly triangular, cutting edge of mandible with four teeth (Fig. 4) [see DuBois 1986; Seifert 2025]	**free-living, non-parasitic species**
–	Individuals much smaller, habitus comparatively gracile, gaster similar in size to mesosoma (ML 0.63–0.81); head broadest at level of posterior margin; anterior border of clypeus lacking teeth, mandibles small, inconspicuous, appearing narrowly strap-like and usually held tightly closed and partly covered by the anterior portion of the clypeus in full face view, cutting edge of mandible (when visible) with fewer than four teeth (Figs 1–3)	**2**
2	Anterior margin of clypeus with small central emargination	**3**
–	Anterior margin of clypeus flat, lacking any central emargination	**4**
3	Central portion of dorsal surface of 1^st^ gastric tergite strongly flattened or concave; antennal scapes and legs with long, erect, reclinate or appressed pilosity; in lateral view, anterior margin of scutum projecting forward as rounded bulge over the pronotum (Fig. 2)	***M.pergandei***
–	Dorsal surface of 1^st^ gastric tergite slightly convex; antennal scapes, head, scutum, legs, and metasoma covered with abundant, long, erect, silvery pilosity; in lateral view, scutum forming a broadly rounded convexity that does not project forward over the pronotum (Fig. 1)	***M.dine* sp. nov.**
4	Erect hairs on scutum and 1^st^ gastric tergite abundant, short, almost stubble-like; propodeum in profile with long, slightly convex dorsal face and much shorter, nearly vertical posterior face (Fig. 3)	***M.talbotae***
–	Erect hairs on scutum and 1^st^ gastric tergite longer, filiform, not stubble-like; propodeum in profile forming an evenly rounded convexity lacking dorsal and posterior faces (Fig. 2)	***M.inquilinum***

### ﻿Species account

#### ﻿ Monomorium dine

Taxon classificationAnimaliaHymenopteraFormicidae

Cover & Rabeling sp. nov.

CD10F498-4D9A-5DD3-BF87-8D1B49B509E7

https://zoobank.org/F09757C2-1F95-4E42-9820-D742B62C262D

##### Diagnosis.

**Figure 1.**
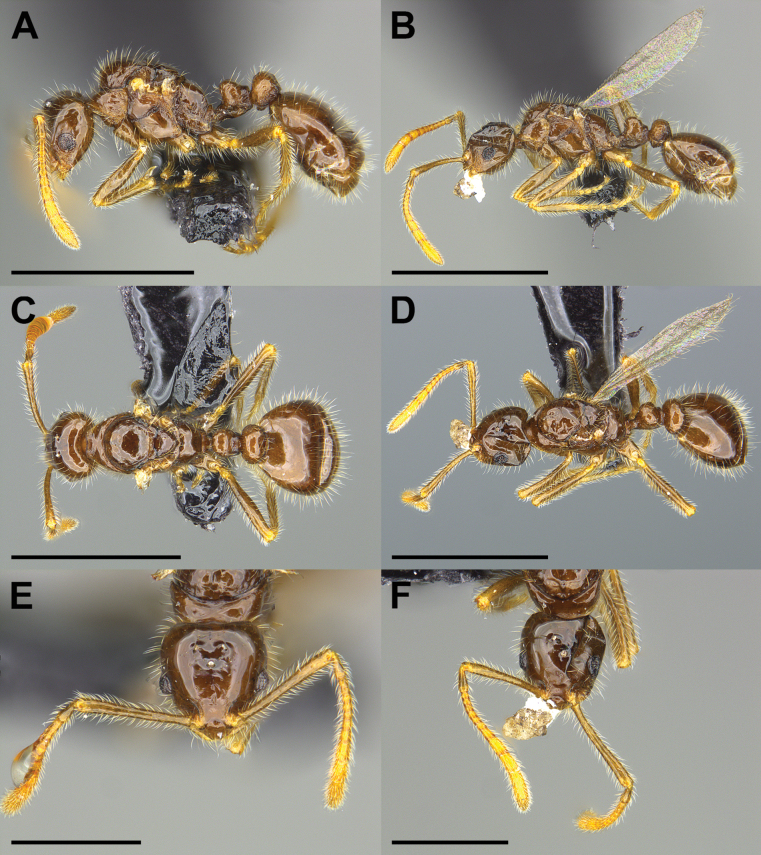
Morphological comparison of the *Monomoriumdine* sp. nov. holotype (**A, C, E**) and paratype (**B, D, F**) females in lateral (**A, B**), dorsal (**C, D**), and full-face (**E, F**) view. The holotype (MCZENT 670596) and the paratype (MCZENT 670597) of *M.dine* were collected in the nest of a free-living *Monomorium* species in the Navajo Nation, New Mexico at Beautiful Mountain. Scale bars: 1 mm (**A–D**); 0.5 mm (**E, F**).

An apparently workerless, inquiline social parasite of a free-living, hitherto undescribed *Monomorium* species. *Monomoriumdine* shows morphological traits of the inquiline syndrome (Fig. 1). Females are miniaturized (i.e., approximately the size of host workers) and have reduced sculpturing compared with the much larger putative host queens (compare Figs 1, 4; see Table 1). Wings are present but are fragile and probably quickly deciduous. Mouthparts are not fully visible, but palp formula appears to be reduced to 1,2. The number of mandibular teeth is reduced to 3. Males are unknown but are probably closely similar in size and habitus to the females.

**Table 1.** Morphological and life history traits characteristic of the inquiline syndrome in New World *Monomorium* ants. Morphological reductions are determined by comparisons to the free-living host species *M.carbonarium* (= *M.minimum*), *M.emarginatum*, and *M.ergatogyna* (= *M.cyaneum*). Traits of the inquiline syndrome modified from Kutter 1968; Wilson 1971; Rabeling et al. 2019; Messer et al. 2020; Prebus et al. 2023; Cover and Rabeling 2024).

	Hosts	Social parasites			
	*M.carbonarium*, *M.emarginatum*, *M.ergatogyna*	*M.pergandei*	*M.inquilinum*	*T.talbotae*	*M.dine*
Worker caste absent	–	+	?(+)	+	? (+)
Multiple egg-laying host queens present (host polygyny)	+	?	?	?	?
Multiple egg-laying parasite queens present in host colony (parasite polygyny)	n/a	?	?	?(+)	?
Parasite queen coexists with host queen (host-queen tolerance)	n/a	?	?	? (+/–)	?
Adelphogamy (inside nest mating)	–	?	?	?	?
Gynaecomorphism	–	+	? (+)	+	? (+)
Fragmented populations, limited geographic distribution	–	+	+	+	+
		(type locality, DC)	(type locality, Estado de Mexico, Mexico)	(CO, MI)	(type locality, NM)
Reduced body size	–	+	+	+	+
		(size of host worker)	(size of host worker)	(size of host worker)	(size of host worker)
Number of antennal segments reduced in females	–	–/+	–	–	–
	(♀: 12)	(♀: 11–12)	(♀: 12)	(♀: 12)	(♀: 12)
Number of antennal segments reduced in males	–	+	?	+	?
	(♂: 13)	(♂:11–12)		(♂: 12)	
Number of maxillary & labial pals (palp formula) reduced in females	–	+	+	+	+(?)
	(♀: 2,2)	(♀: 1,2)	(♀: 1,2)	(♀: 1,2)	(♀: 1,2)
Number of maxillary & labial pals (palp formula) reduced in males	–	+	?	+	?
	(♂: 2,2)	(♂: 1,2)		(♂: 1,2)	
Reduced mandibular dentition	–	+	+	+	+
	(4 teeth)	(3 teeth)	(2 teeth)	(2 teeth)	(3 teeth)
Reduced wings in females	–	+	?	+	+
	(♀: capable of flying)	(♀: wings deciduous)		(♀: wings deciduous)	(♀: wings deciduous)
Reduced wings in males	–	?	?	+	?
	(♂: capable of flying)			(♂: wings deciduous)	

The female of *M.dine* may be easily distinguished from all other New World *Monomorium* social parasites by the abundant, long, erect setae on all body surfaces including the antennal scapes and the legs (Fig. 1). In *M.dine*, the anterior margin of the clypeus has a small central emargination, whereas the emargination is broad in *M.pergandei* and absent in *M.inquilinum* and *M.talbotae*. In addition, *M.dine* can be readily distinguished from *M.pergandei* by the absence of a median impression on the 1^st^ gastric tergite.

##### Description.

***Holotype*** female: HL 0.44, HW 0.43, SL 0.50, ML 0.68, CI 97, SI 117. In full-face view, head narrowly trapezoidal, tapering towards mandibular insertion; head broadest posterior to eyes near posterior corners; dorsal margin straight with corners evenly rounded. Anterior margin of clypeus convex with shallow median impression with tiny central emargination; clypeal carinae absent, median clypeal seta present. Mandibles reduced in size, when closed fitting tightly under the clypeus; apical tooth enlarged, cutting edge with 2 denticles. Antennae with 12 segments; scapes surpassing the dorsal margin of head; with abundant long erect setae. Mouthparts not fully visible, palp formula apparently 1,2. Mesosoma with typical modifications related to wing bearing. Wings appear functional. In lateral view, propodeum forming an evenly rounded convexity, lacking distinct dorsal and posterior surfaces. Propodeal spiracle with small, inconspicuous opening. Opening of metapleural gland tiny, barely visible. In lateral view, petiole with short peduncle and well-rounded dorsal node; postpetiole has a broadly rounded node and a broad ventral tooth. In lateral view, first gastric tergite evenly convex, no trace of median impression. Body surface, antennal scapes, and legs with abundant, long, erect pilosity. Body surfaces smooth, shiny, lacking sculpture except for numerous punctures; lacking pubescence. Color of body uniformly medium brown; appendages slightly lighter, yellowish brown. Paratype females (*n* = 2): HL 0.44, HW 0.41–0.43, SL 0.47–0.50, ML 0.68–0.71, CI 93–97, SI 114–117.

##### Etymology.

This new inquiline social parasite species was discovered on Beautiful Mountain in the Navajo Nation, New Mexico, USA. This new species is named in honor of the Diné People. The species epithet is a noun in apposition.

##### Type Locality.

USA • Navajo Nation, New Mexico, San Juan County, Beautiful Mountain. GPS: 36.5011°N, 108.9672°W; elevation 2544 m. Ponderosa Pine forest; dcbm090711_ant13, Navajo Reservation, under rock. Collected by Gary D. Alpert, 11 July 2009.

##### Type material.

***Holotype*** female (MCZENT 00670596). Two ***paratype*** females (MCZENT 00670595 & 00670597). Holotype and paratypes deposited in the Museum of Comparative Zoology at Harvard University (Cambridge, MA, USA).

##### Discussion and biology.

*Monomoriumdine* is only known from the single collection made at the type locality. Three partially alate females were collected from under a rock, where they were associated with workers of their free-living *Monomorium* host. Unfortunately, the host workers were apparently not collected along with the social parasite. Therefore, the precise identity of the host of *M.dine* remains unknown. However, we have several collections of an undescribed, free-living *Monomorium* species from similar altitudes in neighboring mountain ranges (Fig. 4). In the southwestern United States, *Monomorium* species seem to be distributed altitudinally and overlap between them is uncommon (S.P. Cover unpublished collection data). In this case, the similarity in mountain ranges, altitudes, and habitat types makes us strongly suspect that this undescribed species is also the host of *M.dine.* See Fig. 4 (MCZENT 673296) for an example of this probable host.

**Figure 2.**
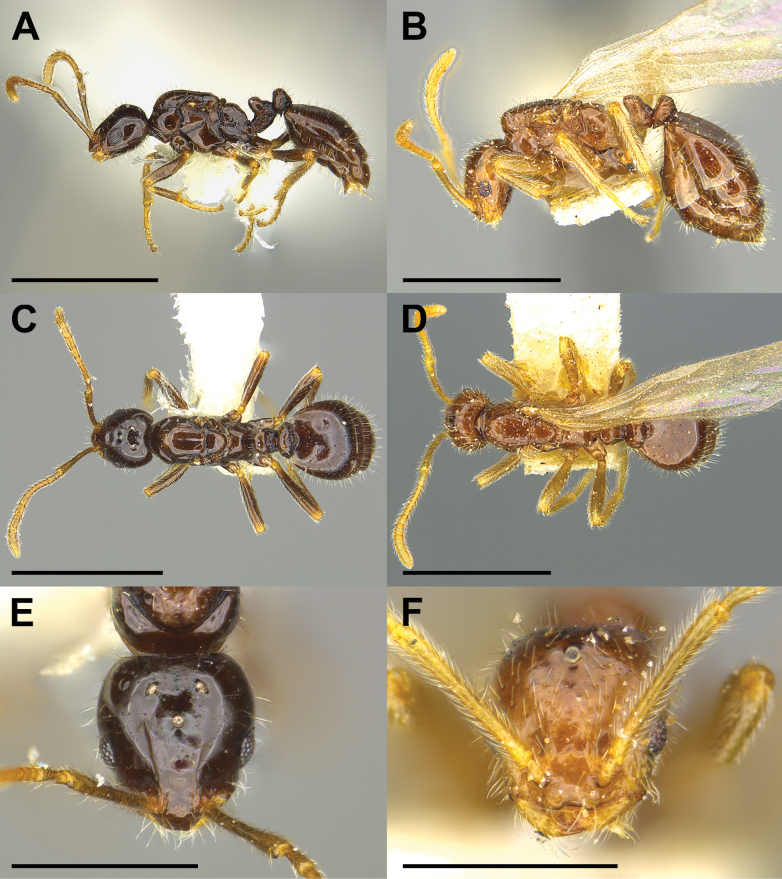
Morphological comparison of the *Monomoriuminquilinum* DuBois holotype (MCZENT 32614) female (**A, C, E**) and a *Monomoriumpergandei* (Emery) paralectotype (MCZENT 815258) female (**B, D, F**) in lateral (**A, B**), dorsal (**C, D**), and full-face (**E, F**) view. Images of *M.pergandei* courtesy of Charles Farnum, MCZ. Scale bars: 1 mm (**A–D**); 0.5 mm (**E, F**).

**Figure 3.**
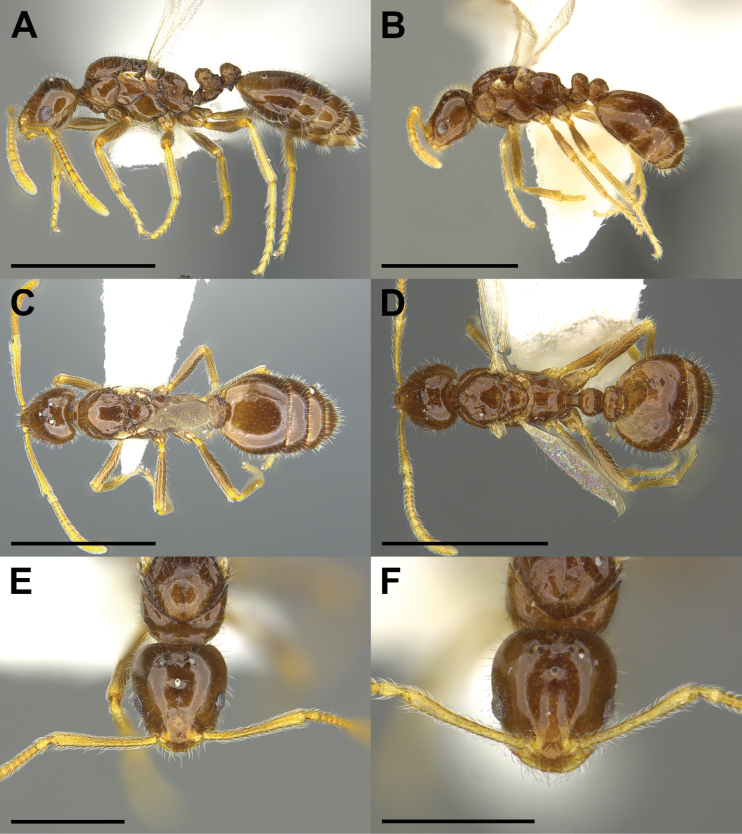
Morphological comparison of *Monomoriumtalbotae* DuBois females from Colorado and Michigan (**A–F**) in lateral (**A, B**), dorsal (**C, D**), and full-face (**E, F**) view. The *M.talbotae* female (MZCENT 655702) depicted in (**A, C, E**) was collected in Colorado (SPC 6704). The *M.talbotae* female (MCZENT 32615) depicted in (**B, D, F**) is the holotype, which was collected by Mary Talbot at the Edwin S. George Reserve in Michigan. Images of the *M.talbotae* holotype courtesy of Charles Farnum, MCZ. Scale bars: 1 mm (**A–D**); 0.5 mm (**E, F**).

**Figure 4.**
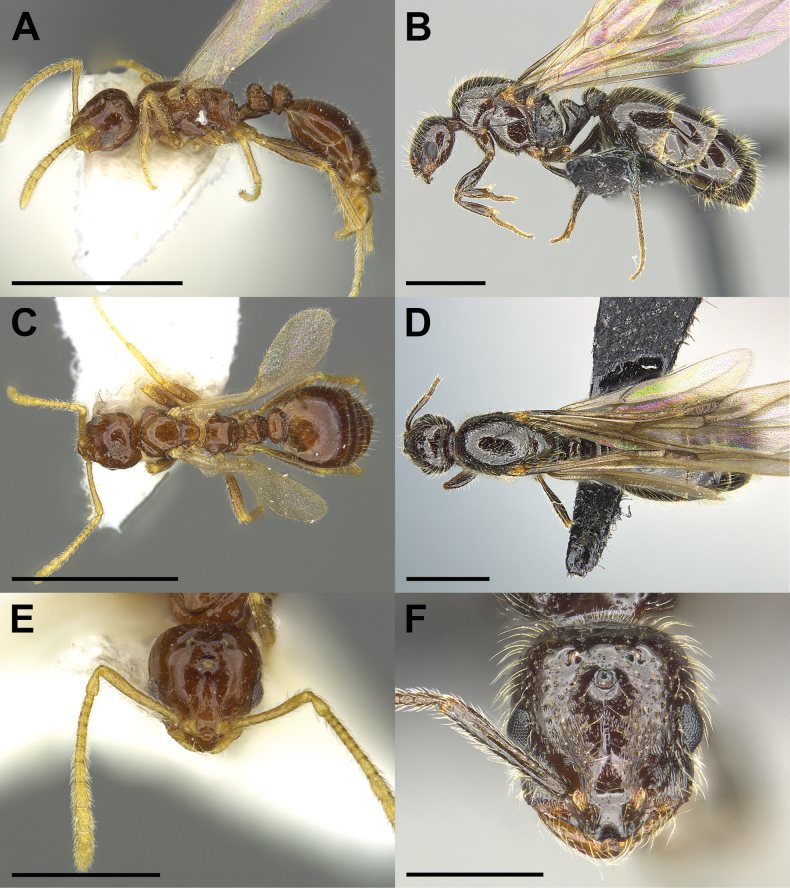
Morphological comparison of the *Monomoriumtalbotae* DuBois paratype male (**A, C, E**) and a *Monomorium* sp. female (**B, D, F**) in lateral (**A, B**), dorsal (**C, D**), and full-face (**E, F**) view. The *M.talbotae* paratype male (MZCENT 522080) was collected at the Edwin S. George Reserve in Michigan. The *Monomorium* sp. female (MCZENT 673296) was collected in the Navajo Nation, Arizona at Black Rock Dike in the Carrizo Mountains and likely represents the host of *M.dine*. Scale bars: 1 mm (**A–D**); 0.5 mm (**E, F**).

Despite the lack of direct evidence concerning the host, the identity of *M.dine* as an inquiline social parasite is secure. It derives from its very close similarity to the other three Nearctic *Monomorium* social parasites (Figs 1–4; see also DuBois 1986). All share characteristics associated with the inquiline syndrome (Kutter 1968; Wilson 1971, 1984; Rabeling et al. 2019; Cover and Rabeling 2024). These include small body size, shiny integument, reduced mandibles, presumably reduced palp formula, the presence of a postpetiolar ventral process (i.e., “Parasitendorn”), the loss of the worker caste, and its exceeding rarity (Table 1). In addition, all four of these species, *M.dine*, *M.inquilinum*, *M.pergandei*, and *M.talbotae* have been collected only in the nests of free-living *Monomorium* species.

## ﻿Discussion

### ﻿Notes on the biology of other North American *Monomorium* inquiline species

***Monomoriumpergandei* (Emery)**

*Monomoriumpergandei* (Fig. 2) was described as belonging to a new genus, *Epoecus* (Emery 1893; see also Brown and Wilson 1957). It has not been seen since. The original description was based on a collection made by Theodore Pergande in Washington, DC on July 12, 1891. As recounted several times in the literature (Wheeler 1910; Creighton 1950; DuBois 1981, 1986), the male and female types were found in a mixed colony with “*Monomoriumminimum*” (= *M.carbonarium*) workers and sexual forms. A fruitless attempt to recollect *M.pergandei* was made by one of us (SPC) in the mid-1980s, but several collections of free-living *Monomorium* were made from Rock Creek Park in the District of Columbia (e.g., MCZENT 00589156, MCZENT 00589199). All are close matches for *M.emarginatum* DuBois, a species currently known only from the northeastern United States. Thus, it is quite likely that the host of *M.pergandei* is *M.emarginatum* and not *M.carbonarium*. Unfortunately, the absence of host queens or workers from the *M.pergandei* type collection makes it impossible to settle the matter conclusively at this time.

The mixed colony Pergande collected did not include workers of *M.pergandei*, but it did include winged queens and males of the host. Pergande observed in an artificial nest that the parasite females attacked and killed some of the host males (Emery 1893, 1895). This puzzling observation caused Wheeler (1910: 498) to suggest that Pergande may have collected two separate colonies nesting in close proximity, one consisting of the social parasite and its host workers while the other contained host workers and alates, and that parasite females attacked the host males because two separate colonies were mixed (see also Creighton 1950: 239–241). While this story is plausible, we may never have sufficient evidence to evaluate the significance of this observation.

#### ﻿*Monomoriumtalbotae* DuBois

*Monomoriumtalbotae* was discovered by Mary Talbot during a population study of “*M.minimum*” (= *M.carbonarium*) at Edwin S. George Reserve in Michigan (Talbot 1979, 2012; DuBois 1981, 1986). See Figs 3, 4 for photos of the holotype queen and a male, respectively. Mary Talbot found three mixed colonies with one containing 6 dealate *M.talbotae* queens, 56 winged queens, and 10 males (col. 30 June 1966). The second colony contained four winged queens and three males (col. 4 July 1966), and the third colony contained 6 dealate queens, one winged queen, and five males (col. 13 July 1966) (Talbot 1979, 2012). Of the three host colonies, none contained individuals identifiable as parasite workers, and one (col. 30 June 1966) contained a single host queen (Talbot 1979, 2012), suggesting that *M.talbotae* might be host-queen tolerant. In addition, the presence of multiple dealate parasite queens suggests that *M.talbotae* may be functionally polygynous. Since Talbot’s original work, two additional occurrences of *M.talbotae* have been documented. A single female specimen from Wexford Co., Michigan has been found in the collection of the USNM. In addition, one of us (SPC) collected *M.talbotae* near Buena Vista, Colorado in 2004 (Fig. 3). The collection data are as follows:

USA, Colorado, Chaffee County, 10.6 km (6.6 miles) south Jct. Rt. 306 (in Buena Vista) on County Rd. 321. GPS: 38°44.54'N, 106°09.71'W; elev. 2621 m (8600′). Open grassy slope with scattered Pinyon (*Pinusmonophylla*) and Ponderosa (*Pinusponderosa*) pines to 9 m (30′) tall. Faint 2.5 cm (1″) diameter crater in bare, sandy soil. Dry conditions. The colony was tiny (<100 ants). No host queen present. Col. S.P. Cover; 09 July 2004; MZCENT 655702; collection code SPC 6704; 30 alate females and 21 host workers.

Several queenright host colonies were collected in the immediate vicinity. The nest queens from Colorado resemble those of *M.emarginatum*, rather than those of *M.carbonarium*. As Mary Talbot’s type series from Michigan does not include queens of the host species, we cannot affirm the identity of the host species at the moment. A forthcoming molecular genetic study will revisit this open question.

#### ﻿*Monomoriuminquilinum* DuBois

*Monomoriuminquilinum* is known from a single dealate female (Fig. 2) collected by Bill Brown on 09 August 1965 in a mixed colony with “*M.cyaneum*” (= *M.ergatogyna*) (DuBois 1981). The nest was found under a rock in a semi desert habitat 83 km south of Queretaro, Estado de Mexico (DuBois 1981). The fact that the *M.inquilinum* queen was collected in a *M.ergatogyna* colony and exhibits morphological modifications of the inquiline syndrome are strong indicators of its social parasitic life history. Nothing else is known about the biology of the species. Note that the identity of this host is also problematic. *Monomoriumcyaneum* was described by Wheeler (1914) from specimens collected in central Mexico, and Seifert (2025) recently synonymized *M.cyaneum* under *M.ergatogyna*. In addition, there appear to be several undescribed *Monomorium* species in the Mexican highlands (R.A. Johnson unpublished collection data). Because none of Brown’s host specimens are extant, the identity of the *M.inquilinum* host remains speculative.

### ﻿Concluding remarks

The Nearctic *Monomorium* inquiline species are a particularly peculiar group of ant social parasites. Though very little or nothing is known about the life histories of any of the four species in this group, the fragmentary evidence we have is consistent with all four being workerless, inquiline social parasites of free-living *Monomorium* species. Beyond that all other interpretations are uncertain. Mary Talbot’s natural history observations suggest that *M.talbotae* is polygynous and may possibly be host-queen tolerant (Talbot 1979, 2012). Our Colorado collection of *M.talbotae* (SPC 6704), however, lacked both a host queen and host brood, and was from a local host population in which every other nest excavated contained one to several host queens (S.P. Cover, unpublished collection data). These observations imply that the Colorado population of *M.talbotae* may be host-queen intolerant. Pergande’s observations of attacks by *M.pergandei* females on host males are perhaps suggestive (Emery 1895). If these parasites are host-queen intolerant, they may belong to the class of social parasites that preferentially enter queenless host nests. It is hard to imagine these parasite females attacking much larger host queens successfully with their tiny, strap-like mandibles. However, the sting is well developed in parasite females (Figs 2, 3), and Pergande’s observations of fighting suggest they may be capable of attacking host queens using their stings.

Host identity is a problem with all North American *Monomorium* social parasites. Historically, the taxonomy of the free-living host species has been confused. Until recently, the oldest taxon in the group was *M.minimum* (Buckley), described 1867 and no types are extant (Buckley 1867). In subsequent years, additional new taxa were described, in large part due to uncertainty regarding the identity of the “true” *M.minimum.* This resulted in confusion regarding all the taxa and the lumping of many collections from all over the distribution range under the “umbrella” name “*M.minimum*.” DuBois (1986) made a pioneering attempt to sort out the tangle, but problems remained. Recently, Seifert (2025) revised the taxonomy of the *M.carbonarium* group and designated *M.minimum* a junior synonym of *M.carbonarium*, thus changing the name of the species group. Seifert (2025) recognized the following species as taxonomically valid: *M.carbonarium* (Smith), *M.compressum* Wheeler, *M.ebeninum* Forel, *M.emarginatum* DuBois, *M.ergatogyna* Wheeler, *M.lorenzoi* Seifert, *M.marjoriae* DuBois, and *M.viridum* Brown. Our forthcoming molecular genetic study of North American *Monomorium* species will revisit this taxonomic puzzle.

Additional natural history observations are needed to better understand the biology and life history of these social parasites. A hint that may increase the possibility of new collections is that, as for many inquiline ants, the chances of discovery are much higher when alate parasites are present in the nest. Many dealate inquiline females are as small or smaller than host workers and are similar in coloration as well, making them very difficult to spot during collecting. In contrast, alate inquiline sexual forms are comparatively conspicuous because they are generally much smaller and more delicate in habitus than those of the host. They may also be present at times when host sexual forms are absent. Any colony with unusually small, winged ants present deserves careful investigation. We hope that new collections of these *Monomorium* inquilines will expand our knowledge of this interesting and presently mysterious group.

## Supplementary Material

XML Treatment for Monomorium dine

## ﻿Additional information

### Author ORCIDs

Stefan P. Cover https://orcid.org/0009-0000-1911-0283

Christian Rabeling https://orcid.org/0000-0003-1292-0309

### Data availability

All of the data that support the findings of this study are available in the main text.
